# Evaluating Health Risks from Inhaled Polychlorinated Biphenyls: Research Needs for Addressing Uncertainty

**DOI:** 10.1289/ehp.1408564

**Published:** 2014-10-10

**Authors:** Geniece M. Lehmann, Krista Christensen, Mark Maddaloni, Linda J. Phillips

**Affiliations:** 1 National Center for Environmental Assessment, Office of Research and Development, U.S. Environmental Protection Agency, Research Triangle Park, North Carolina, USA; 2 National Center for Environmental Assessment, Office of Research and Development, U.S. Environmental Protection Agency, Washington, DC, USA; 3 Region 2, U.S. Environmental Protection Agency, New York, New York, USA

## Abstract

Background: Indoor air concentrations of polychlorinated biphenyls (PCBs) in some buildings are one or more orders of magnitude higher than background levels. In response to this, efforts have been made to assess the potential health risk posed by inhaled PCBs. These efforts are hindered by uncertainties related to the characterization and assessment of source, exposure, and exposure-response.

Objectives: We briefly describe some common sources of PCBs in indoor air and estimate the contribution of inhalation exposure to total PCB exposure for select age groups. Next, we identify critical areas of research needed to improve assessment of exposure and exposure response for inhaled PCBs.

Discussion: Although the manufacture of PCBs was banned in the United States in 1979, many buildings constructed before then still contain potential sources of indoor air PCB contamination. In some indoor settings and for some age groups, inhalation may contribute more to total PCB exposure than any other route of exposure. PCB exposure has been associated with human health effects, but data specific to the inhalation route are scarce. To support exposure–response assessment, it is critical that future investigations of the health impacts of PCB inhalation carefully consider certain aspects of study design, including characterization of the PCB mixture present.

Conclusions: In certain contexts, inhalation exposure to PCBs may contribute more to total PCB exposure than previously assumed. New epidemiological and toxicological studies addressing the potential health impacts of inhaled PCBs may be useful for quantifying exposure–response relationships and evaluating risks.

Citation: Lehmann GM, Christensen K, Maddaloni M, Phillips LJ. 2015. Evaluating health risks from inhaled polychlorinated biphenyls: research needs for addressing uncertainty. Environ Health Perspect 123:109–113; http://dx.doi.org/10.1289/ehp.1408564

## Introduction

Polychlorinated biphenyls (PCBs) consist of two linked benzene rings in which one or more of the hydrogen atoms have been replaced by chlorine atoms. Ten positions are available for substitution; there are 209 PCB congeners defined by the number and placement of the chlorine atoms. Mixtures of PCB congeners were manufactured in the United States from 1929 to 1977 for use as coolants and lubricants in transformers, capacitors, and other electrical equipment [Agency for Toxic Substances and Disease Registry (ATSDR) 2000]. They were also used in building construction materials as additives to elastic sealants, caulking, grouts and paints, and flame retardant coatings of acoustic ceiling tiles (Erickson and Kaley 2011; Heinzow et al. 2007). Unfortunately, the unique chemical properties of PCBs that made them useful in commercial applications (e.g., thermal stability, resistance to acids and bases, low water solubility) also contributed to their resistance to degradation, bioaccumulation in terrestrial and aquatic food chains, long-range transport, and toxicity (ATSDR 2000). Although the commercial manufacture of PCBs was banned in the United States in 1979, they continue to be released into the environment through the use and disposal of PCB-containing products.

In 2004, Herrick et al. (2004) conducted a study of 24 buildings in and around Boston, Massachusetts. Exterior caulk samples from eight buildings contained PCB concentrations high enough [range, 70–36,200 ppm (parts per million); mean, 15,600 ppm] to require the material to be treated as PCB bulk product waste [U.S. Environmental Protection Agency (EPA) 2015]. MacIntosh et al. (2012) reported on an elementary school with PCB-containing caulk (range, 1,830–29,400 ppm) that had a mean PCB indoor air concentration > 500 ng/m^3^: orders of magnitude greater than typical background concentrations in ambient urban air [1–10 ng/m^3^ (ATSDR 2000)]. Similar indoor air PCB concentrations have been reported for other buildings constructed with PCB-containing caulk (Heinzow et al. 2007; Kohler et al. 2005; Williams et al. 1980).

Caulk is only one of several potential sources of indoor air PCB contamination (Thomas et al. 2012). Additional primary sources (i.e., those that were manufactured containing PCBs or had PCBs added during construction) that might currently be found in buildings include window glazing, fluorescent light ballasts, ceiling tile coatings, and other materials such as paints or floor finishes. Secondary sources of PCBs may also contribute to elevated indoor air PCB concentrations. Secondary sources are defined here as materials that become contaminated through absorption from direct contact with primary PCB sources, or through absorption of PCBs in the indoor air that have been emitted by primary sources. Secondary sources may include paints, mastics, ceiling tiles, flooring, and wall boards.

Caulk and other building materials containing PCBs were widely used from the 1950s through the 1970s. PCBs in fluorescent light ballasts were discontinued in the late 1970s, but many buildings throughout the United States that were constructed before 1979 may still have fluorescent lighting fixtures that contain PCBs and/or PCB residues from leaking or previously burned-out ballasts (U.S. EPA 2013). Thus, human inhalation exposure to PCBs may be more widespread than previously assumed.

## Discussion

Humans can be exposed to PCBs via ingestion, inhalation, or dermal contact. Consumption of contaminated food has historically been considered the major route of exposure among the general population, with fatty foods (i.e., fish, meat, dairy products) being the major contributors to dietary exposure (ATSDR 2000). Based on data from the Food and Drug Administration Total Diet Study (Pennington 2000), which evaluates ready-to-eat foods to determine the dietary intake of selected contaminants, dietary exposures to PCBs have declined over the last several decades (e.g., from 27 ng/kg-day in 1978 to 2 ng/kg-day in 1997 for adults) (ATSDR 2000).

Inhalation may be an important contributor to total PCB exposure. To evaluate its contribution to total exposure, we estimated potential total and route-specific background PCB indoor and outdoor exposures (see Supplemental Material, Table S1). We estimated exposure for ingestion of soils and dusts, ingestion of food, dermal contact, and inhalation for children (i.e., ages 2–3 years and 6–12 years) and adults using average PCB exposure concentrations reported in the scientific literature (Harrad et al. 2009; Priha et al. 2005) and exposure factors from the U.S. EPA’s *Exposure Factors Handbook* (U.S. EPA 2011).

For children ages 2–3 years and 6–12 years and adults, respectively, total PCB exposures were 6.9, 4.7, and 3.2 ng/kg-day (see Supplemental Material, Table S1). The estimates suggest that the extent of inhalation exposure to PCBs in some indoor settings may be at least as large as typical dietary exposure. Inhalation of indoor air was estimated to account for 60.8, 50.5, and 34.6% of total exposure, whereas diet accounted for 28.9, 42.7, and 62.8% of total exposure for children ages 2–3 years and 6–12 years and adults, respectively. The contributions to total exposure from all other pathways, including outdoor air, were relatively small. The exposures estimated here were based on background PCB concentrations. Total exposures in PCB-contaminated buildings would be expected to be higher, and the contributions from the various exposure pathways would likely differ from those reported here, with inhalation potentially accounting for an even greater proportion of the overall exposure. These results suggest the need to consider inhalation when evaluating the overall human health risk from exposure to PCBs in some environments.

To characterize the health risk associated with PCB exposure, information is needed on relevant health outcomes and their exposure–response relationships. This information may come from human or laboratory animal studies. Many such studies have shown that chronic oral PCB exposure is associated with both cancer and noncancer health effects (ATSDR 2000; Lauby-Secretan et al. 2013), but there is a profound lack of data to support exposure and exposure–response assessment for inhaled PCBs.

Three intermediate-duration studies in animals provide information on the effects of inhaled PCBs. Treon et al. (1956) observed histopathologic lesions in livers of rats, mice, rabbits, and guinea pigs exposed to 1.5 × 10^6^ ng/m^3^ of a mixture of PCB congeners volatilized from Aroclor 1254 for 7 hr/day, 5 days/week for a total study duration of 213 days. Casey et al. (1999) exposed adolescent male Sprague-Dawley rats to an atmosphere containing 900 ng/m^3^ of a PCB mixture volatilized from Aroclor 1242 via whole-body inhalation for 23 hr/day for 30 days. Effects included significant histopathological changes in the thyroid and thymus, increases in serum thyroid hormone concentrations, and a significant decrease in normal exploratory behavior. Notably, the inhalation exposure level tested was within the range of concentrations observed in some public buildings. Hu et al. (2012) observed minimal toxicity in female Sprague-Dawley rats exposed via nose-only inhalation to 5.2 × 10^5^ ng/m^3^ of a PCB mixture developed to mimic the congener profile of air in Chicago, Illinois, for an average of 1.6 hr/day, 5 days/week for 4 weeks. Many toxicological end points were investigated, but only minimal responses were observed. Although relative thymus weight was 12.5% lower in the PCB-exposed group, the difference was not statistically significant. In contrast with Casey et al. (1999), Hu et al. (2012) did not report histopathological effects in the thyroid or measure changes in serum thyroid hormone concentrations or neurobehavioral end points. Differences in the exposure methods used by these two studies (i.e., whole-body vs. nose-only), the PCB congener mixtures tested, and the number of hours per day that the rats were exposed (i.e., 23 vs. 1.6) make it difficult to interpret differences in the results.

Of the three toxicological studies of PCB inhalation exposure described above, two (Casey et al. 1999; Treon et al. 1956) reported notable health effects, identifying inhalation exposure to PCBs as a potential health hazard. However, for human health risk assessment, preferred exposure–response data are those which demonstrate a clear, quantifiable association between exposure and response measured across multiple exposure levels (U.S. EPA 2002). In the Treon et al. (1956) study, several experimental and control-group animals died from extraneous causes, including an epidemic of pneumonia, which could confound the association between PCB exposure and liver histopathology. Also, there may be some uncertainty in the exposure estimate reported by Casey et al. (1999) because of this study’s use of whole-body inhalation exposure (vs. nose-only). As stated by the study authors, whole-body exposure was chosen over nose-only exposure to prevent stress to the animals that would result from restraint for 23 hr/day; such stress could affect the behavioral, endocrine, and immunological end points evaluated. However, a disadvantage of whole-body exposure is that possible deposition of test article on animal fur or skin may result in additional unquantified oral exposure to the animals as a result of grooming behavior. Furthermore, Casey et al. (1999) did not report complete exposure information including breathing zone concentrations and evidence supporting a uniform distribution of chemical within the exposure chamber.

Although studies investigating health effects from inhaled PCBs are limited, the collective results of those studies suggest potential to result in adverse health effects. Robust exposure–response information is important to characterize the risk of inhaled PCBs; thus, additional research is critical to support exposure–response assessment. Needed studies include epidemiological and/or laboratory animal-based investigations with well-characterized PCB exposure by inhalation. Initial studies might focus on health end points known to be sensitive to disruption by PCB exposure (e.g., developmental neurotoxicity, immunotoxicity, and changes in thyroid hormone levels) (ATSDR 2000). Examples of the studies needed, including important study design considerations, are provided as Supplemental Material (Appendix S1).

Because PCBs exist in the environment as mixtures, careful consideration should be given to which congeners should be measured to monitor exposure. In some cases, biomonitoring data or information on PCB-containing products manufactured or used in a specific study setting can guide this choice. However, PCB body burden reflects exposure integrated over all routes and pathways (including inhalation and diet), over a prolonged time interval. Consequently, it is not generally possible to determine how much of a person’s body burden may be attributable to exposure only from inhalation during a specified period of time.

Furthermore, as reviewed by Hansen (1998), many epidemiological studies have assessed exposure based on the analysis of limited subsets of PCB congeners selected because of their toxicological similarity to dioxin, their relative occurrence in biological samples, and/or the ease with which they can be detected using a given analytical method. In most cases, these subsets favor congeners found at high levels in food chains, neglecting congeners found in indoor and outdoor air. And, many of the congeners commonly found in air have relatively short biological half-lives; therefore, even if these congeners are analyzed, associations between exposure and effect may be missed if exposure assessment is based solely on long-term PCB body burden.

Despite these limitations, in some cases where one exposure source is dominant, it may be possible to disentangle the pathways of exposure contributing to PCB body burden. Several studies have used biomonitoring to evaluate PCB exposure for persons spending time in buildings where PCBs are known to be present (e.g., Herrick et al. 2011; Meyer et al. 2013). In general, patterns of exposure differ between “exposed” and “unexposed” persons, with exposed persons having higher proportions of lower-chlorinated congeners (i.e., congeners containing ≤ 4 chlorines). Physiologically based pharmacokinetic models describing the kinetic properties of inhaled PCB congeners with varying numbers of chlorines may facilitate the use of biomonitoring data for assessing inhalation exposures. Human and animal studies can support the development of such models by measuring individual PCB congeners and metabolites in biological samples taken at various time points relative to exposure. There has also been recent progress in characterizing metabolites of some PCB congeners found predominantly in air (Dhakal et al. 2012). After careful validation, it may be possible to use measures of certain PCB congeners or their metabolites as useful biomarkers of inhalation exposure to PCBs; but in the meantime, it is important for epidemiological studies to characterize exposure based on PCB concentrations in air using analytical methods that can detect a wide range of congeners (e.g., Hu et al. 2008).

In a general population setting, one straightforward approach would be to have study participants use personal air monitors to measure PCB exposure levels. Another approach would be similar to those described above (see Supplemental Material, Table S1) and in a report by Thomas et al. (2012), in which information on PCB levels in air in different locations (e.g., inside an office building, inside a residence, outside) was combined with information on each individual’s time spent in monitored areas (e.g., a person’s work schedule and time spent at home). Adjustments for inhalation rate may be made for factors such as sex, age, and exertion level to more accurately quantify the degree of exposure (U.S. EPA 2011). It may also be useful to gather data to compare dietary PCB exposures in inhalation-exposed and reference populations.

Initially, epidemiological studies should investigate associations between PCB inhalation exposure and key health effects identified from studies of PCB body burden, including the following: changes in serum thyroid hormone levels, increased susceptibility to infection, decreased antibody responses to immunization, and cognitive effects in children (Carpenter 2006). Populations of interest include adults and children directly exposed to PCB-contaminated air, and children born to women exposed to PCBs by inhalation before or during pregnancy and lactation. Repeated exposure assessment at various time points in both children and their mothers during (or even before) pregnancy may reveal associations between key health effects and PCB exposures during sensitive periods of development (Makris et al. 2008). PCB exposures before pregnancy may be important because many PCB congeners accumulate in body lipids and may be transferred to an infant during pregnancy or through breastfeeding. As described by Lehmann et al. (2014), this toxicokinetic property of PCBs may also have important implications for developmental exposure studies in animals, which may benefit from the inclusion of an exposure period before mating and gestation.

Relevant end points (e.g., changes in serum thyroid hormone levels, neurodevelopmental effects, and immunosuppression) can be measured in both humans and animals. For example, relevant neurodevelopmental end points can be tested using assays of schedule-controlled behavior, attention and associative processing, spatial learning, and behavioral inhibition (Rice and Barone 2000). The most relevant assays for determining potential immunotoxicity hazard are those measuring disease resistance or immune function (e.g., antibody response to antigen, and natural killer cell activity) (International Programme on Chemical Safety 2012). Gathering data across species and showing similar patterns of effects for concordant outcomes provides important support for the use of animal exposure–response data in human health risk assessment.

A chronic or developmental exposure study in animals exposed to PCBs by inhalation would be an improvement over the intermediate-duration data that are currently available to support exposure–response assessment for inhalation exposure to PCBs. Because offspring exposure occurs by maternal transfer first via the placenta and then through milk, assessment of total offspring exposure can be facilitated by measuring PCB body burden directly in the offspring (Lehmann et al. 2014). This is especially important because of the above-mentioned tendency of PCBs to accumulate in adipose tissue before pregnancy; these established maternal PCB stores can contribute to offspring exposure during pregnancy and lactation, resulting in higher exposures to the offspring than would occur only with concurrent maternal PCB exposure by inhalation or other routes. Important data might also be gleaned from a developmental study using oral exposure if *a*) the administered PCB mixture were patterned after a congener profile observed in air (e.g., Zhao et al. 2010), and *b*) toxicokinetic data were collected to support route-to-route extrapolation.

An additional factor important to consider in PCB inhalation study design is the specific method used to deliver PCBs to the animals (i.e., whole-body or nose-only exposure). As mentioned above as a potential point of concern with the study by Casey et al. (1999), whole-body exposure to volatilized PCBs may allow for some additional unquantified oral exposure to occur as a result of animal grooming behavior. However, there are also disadvantages of nose-only exposure. The restraint required for nose-only exposure is associated with stress-related responses that might interfere with the detection of exposure-related responses. Also, to prevent undue stress to the animals, nose-only exposure must be limited to only a few (e.g., 4–6) hours per day. And nose-only exposure systems accommodate a limited number of animals at one time, which may reduce the total number of animals that can be used in a single study.

Another improvement to the existing exposure–response data could come from a study testing three or more PCB concentrations to support benchmark dose modeling, which is preferred over the NOAEL/LOAEL (no observed adverse effect level/lowest observed adverse effect level) approach to exposure–response assessment (U.S. EPA 2012). Although a study of chronic duration may be preferred when using data to support human health risk assessment for chronic exposures, even a study of subchronic duration would be an important contribution to the existing PCB inhalation database if it were to measure health effects at three or more exposure levels.

In addition to the study design elements described above, there are additional factors to consider when planning studies of PCB inhalation exposure. A PCB mixture’s physicochemical properties and toxicity are related to the degree of congener chlorination and the specific congeners that constitute the mixture. For example, lower-chlorinated congeners are much more volatile than higher-chlorinated congeners. Thus, the PCB congener profile found in air may not match the congener profile found in source material (e.g., caulk) because of differences in congener volatility. For exposure assessment, it is important to determine the congener composition of the PCB mixture in air to which humans or animals are exposed.

In an inhalation study conducted in animals, one goal might be to use a volatilized PCB mixture with a congener profile most similar to a “typical” human inhalation exposure. However, although some progress has been made in characterizing the congener profiles of PCBs in air from different geographical regions and in different contexts, a great deal of variability has been observed (Persoon et al. 2010). For example, congener profiles of air in Chicago were dominated by congeners with ≤ 5 chlorines (Zhao et al. 2010), but congener profiles of outdoor air in Rice Creek, New York, contained large percentages of congeners with 6 or 7 chlorines (Chiarenzelli et al. 2001). In Germany, researchers noted that rooms contaminated with PCB-containing caulk contained a much lower proportion of “dioxin-like” congeners [i.e., PCBs 77, 81, 105, 114, 118, 123, 126, 156, 157, 167, 169, and 189 (Van den Berg et al. 2006)] in air than rooms with acoustic ceiling tiles treated with PCB-containing flame retardants (Heinzow et al. 2007). Thus, different congener patterns in indoor air may exist in buildings with different PCB sources and conditions.

To support both exposure and exposure–response assessment, additional work is needed to measure specific congeners in the air of buildings with PCB sources to better characterize and understand the range of relevant inhalation mixtures and specific congener concentrations. It is likely that toxicological data from a number of different mixtures of inhaled PCBs will be needed to adequately assess human health risk across a number of relevant exposure scenarios. Preferably, both epidemiological and toxicological studies of PCB inhalation would characterize PCB congener profiles in the exposure atmosphere to facilitate comparison of results across studies.

For toxicological testing, additional work is needed to develop and characterize PCB mixtures in air. Previous studies of the health effects of environmental PCBs have used synthetic mixtures developed from individual PCB congeners (e.g., Rice and Hayward 1997) or extracts of contaminated environmental media (e.g., Li and Hansen 1996). More recently, researchers have found some success in formulating environmental PCB mixtures by combining multiple commercial mixtures (Kostyniak et al. 2005; Zhao et al. 2010). Most notably, Zhao et al. (2010) developed a PCB mixture resembling the average congener profile in Chicago air by combining Aroclors 1242 and 1254. An atmosphere generated from this mixture was used by Hu et al. (2012) to investigate PCB inhalation toxicity in female rats. Although these studies reveal a promising initial approach for developing and testing inhaled PCB mixtures, more work is needed. For example, achieving vapor-phase levels of higher-chlorinated PCB congeners in a laboratory setting that match those in environmental air samples has proven to be difficult (Hu et al. 2012). Furthermore, some PCB congeners present in environmental media, including air, are not found in commercial mixtures (e.g., PCB-11) (Hu et al. 2008), suggesting the possible need to supplement the test mixtures used in toxicological studies with any missing congeners.

The relationship between a PCB mixture’s congener content and its toxicity has been shown to vary depending on the specific health effect under investigation. Based on oral PCB exposure studies, mixtures with larger percentages of both higher-chlorinated and dioxin-like congeners are the most potent for inducing hepatocellular neoplasms, hepatic histopathological changes, and thyroid follicular-cell hyperplasia in rodents (Mayes et al. 1998). In contrast, these mixtures have not been found to be more potent than lower-chlorinated PCB mixtures in immunotoxicity and neurotoxicity assays (Harper et al. 1995; Seegal et al. 1991). Because the same type of complexity is expected for PCB inhalation, investigating a wide range of health effects will be important before drawing conclusions regarding the relative toxicity of a particular inhalation exposure mixture.

PCB mixtures that have been extensively tested in studies of oral exposure may be very different in congener composition than mixtures found in air. Compared with higher-chlorinated congeners, the lower-chlorinated PCBs commonly found in air are more likely to metabolize to reactive hydroquinones or quinones, which may contribute to genotoxicity and other forms of cellular damage (Robertson and Ludewig 2011). Some lower-chlorinated congeners, or their metabolites, have been reported to be active in neurotoxicity, estrogenicity, and genotoxicity assays (Hansen 1998; Robertson and Ludewig 2011). The congeners most potent at depleting dopamine in pheochromocytoma cells (PCB-4) and increasing phorbol ester binding in cerebellar granule cells (PCB-52) are lower-chlorinated congeners detected at high levels in Chicago air (Hansen 1998; Zhao et al. 2010). Another lower-chlorinated congener (PCB-3) has been shown to increase the mutation frequency in the liver of male Big Blue rats, an observation supported by *in vitro* studies in which metabolites of this congener have induced genetic mutations, chromosome breaks, chromosome loss, and polyploidization (Robertson and Ludewig 2011). There may also be differences in tissue distribution or metabolism of PCBs between the inhalation and oral routes of exposure. For these reasons, it is quite possible that inhalation studies will reveal additional toxicological end points that are particularly susceptible to PCB exposure by inhalation.

## Conclusions

Highly elevated indoor air PCB concentrations have been observed in some buildings. PCB-containing caulk, fluorescent light ballasts, or other PCB sources may be found in any building, public or private, constructed before 1979 (e.g., schools, office buildings, hospitals, houses of worship, residences, and many others), contributing to elevated indoor air PCB concentrations (Meyer et al. 2013; Schettgen et al. 2012). However, because so few studies of PCB inhalation toxicity have been published, the ability to confidently assess health risk to the occupants of these buildings is limited. Additional inhalation exposure and toxicity information for humans and/or animals exposed to PCBs by inhalation would better inform the assessment of PCB risks from this exposure route.

## Supplemental Material

(298 KB) PDF
