# Supplementary material for: Evaluating Health Risks from Inhaled Polychlorinated Biphenyls: Research Needs for Addressing Uncertainty
Source: Environ Health Perspect. 2014 Oct 10;123(2):109–13. doi: 10.1289/ehp.1408564 (PMC4314250; doi:10.1289/ehp.1408564)
Supplement: (298 KB) PDF [file ehp.1408564.s001.508.pdf]

Supplemental Material

Evaluating Health Risks from Inhaled Polychlorinated Biphenyls: Research Needs for Addressing Uncertainty

Geniece M. Lehmann, Krista Christensen, Mark Maddaloni, and Linda J. Phillips

Table S1. Exposure equations, input assumptions, estimated exposures (ng/kg-day), and percentages of total exposures for 3 age groups.

Exposure scenario	Exposure equation	Total exposure[a] (2 to <3 yrs)	Total exposure[a] (6 to <12 yrs)	Total exposure[a] (adult)	Percent total exposure (2 to <3 yrs)	Percent total exposure (6 to <12 yrs)	Percent total exposure (adult)
Dust ingestion	$ADD_{dust} = (C_{dust} \times IngR_{dust} \times CF \times Abs_{dust-soil}) / BW$	0.57	0.25	0.05	8.3%	5.3%	1.6%
Soil ingestion	$ADD_{soil} = (C_{soil} \times IngR_{soil} \times CF \times Abs_{dust-soil}) / BW$	0.11	0.05	0.01	1.6%	1.0%	0.2%
Indoor Inhalation	$ADD_{inhalation-indoor} = (C_{air-indoor} \times IR \times F_{indoors} \times CF_1 \times Abs_{air}) / BW$	4.2	2.4	1.1	60.8%	50.5%	34.6%
Outdoor inhalation	$ADD_{inhalation-outdoor} = (C_{air-outdoor} \times IR \times F_{outdoors} \times CF_1 \times Abs_{air}) / BW$	0.017	0.017	0.019	0.3%	0.4%	0.6%
Dermal contact	$ADD_{dermal} = (C_{dust} \times Ad \times SA \times CF \times Abs_{dermal}) / BW$	0.010	0.0080	0.0037	0.1%	0.2%	0.1%
Dietary intake	ADD_{food}	2.0	2.0	2.0	28.9%	42.7%	62.8%
**Total all pathways**	$ADD_{dust} + ADD_{soil} + ADD_{inhalation-indoor} + ADD_{inhalation-outdoor} + ADD_{dermal} + ADD_{food}$	6.9	4.7	3.2	100%	100%	100%

[a]Total exposure was calculated by adding the exposures from the individual exposure pathways and multiplying by 1,000 to convert to units of ng/kg-day.

Parameter Definitions and Input Assumptions for preschool (2 to <3 year) and elementary (6 to <12 year) children, and adults:

$Abs_{dust-soil}$ = Relative absorption factor for soil or dust ingestion [unitless; estimated as soil oral absorption fraction/food oral absorption fraction = 0.5/0.8. Absorption fraction is assumed to be 50% for soil and 80% for food based on data for dioxins (U.S. EPA 2003)].

Abs_{air} = Relative absorption factor for inhalation [unitless; absorption of inhaled PCBs is assumed to be the same as for ingested PCBs based on information for dioxins indicating very high inhalation absorption (U.S. EPA 2003)].

Abs_{dermal} = Relative absorption factor for dermal contact [unitless; estimated as dermal absorption fraction/food oral absorption fraction = 0.07/0.8; dermal absorption fraction for PCBs in soil is assumed to be 7%, based on Roy et al. (2009) and 80% for food based on data for dioxins (U.S. EPA 2003)].

Ad = Dust to skin adherence [0.005 mg/cm^2-day for children and 0.003 mg/cm^2-day for adults based on data from U.S. EPA (2011)]

ADD_{dust} = Average daily dose from ingestion of dust (µg/kg-day).

ADD_{soil} = Average daily dose from ingestion of soil (µg/kg-day).

$ADD_{inhalation\text{-}indoor}$ = Average daily dose from inhalation of indoor air (µg/kg-day).

ADD_{dermal} = Average daily dose from dermal contact with indoor dust (µg/kg-day).

ADD_{food} = Average daily dose from food (0.002 µg/kg-day); based on FDA Total Diet Study data for foods collected in 2003 (FDA 2014); data for the total population used.

BW = Body weight [13.8, 31.8 and 80 kg for ages 2 to <3 yrs, 6 to <12 yrs, and adults, respectively (U.S. EPA 2011)].

C_{dust} = Concentration of PCBs in dust [0.22 µg/g; based on data from Harrad et al. (2009); mean total PCB concentration in dust samples collected from 20 homes in Austin, TX].

C_{soil} = Concentration of PCBs in soil [0.05 µg/g; based on data from Priha et al. (2005); mean urban background concentration of PCBs in soil samples collected from parks in Helsinki, Finland; data for U.S. background concentrations are limited].

$C_{air\text{-}indoor}$ = Concentration of PCBs in indoor air [6.9 ng/m^3; based on data from Harrad et al. (2009); mean total PCB concentration in air from 10 homes in Toronto, Canada].

$C_{air\text{-}outdoor}$ = Concentration of PCBs in outdoor air (0.5 ng/m^3; based on estimates in Harrad et al. (2009) from data in Motelay-Massei et al. (2005); average total PCBs in outdoor air in Toronto, Canada)

CF = Conversion factor (g/1,000 mg)

CF_1 = Conversion factor (µg/1,000 ng)

$F_{indoors}$ = Fraction of time spent indoors (unitless; 0.95, 0.91 and 0.80 assumed for ages 2 to <3 yrs, 6 to <12 yrs, and adults, respectively)

$F_{outdoors}$ = Fraction of time spent outdoors (unitless; 0.05, 0.09 and 0.20 assumed for ages 2 to <3 yrs, 6 to <12 yrs, and adults, respectively)

$IngR_{dust}$ = Dust ingestion rate (60 mg/day for children and 30 mg/day for adults (U.S. EPA 2011))

$IngR_{soil}$ = Soil ingestion rate (50 mg/day for children and 20 mg/day for adults (U.S. EPA 2011))

IR = Inhalation rate (8.9, 12.0 and 15.9 m^3/day for ages 2 to <3 yrs, 6 to <12 yrs, and adults, respectively (U.S. EPA 2011))

SA = Skin contact area (1,365, 2,554 and 4,991 cm^2 for ages 2 to <3 yrs, 6 to <12 yrs, and adults, respectively; based on data from U.S. EPA (2011); assumes contact with the hands, 55% of the arms, and 39% of the legs)

Appendix S1. PCB Inhalation Data Gaps and Example Studies to Address Those Gaps

Exposure-response data from human populations exposed to PCBs by inhalation

Epidemiological studies in which (1) the degree of inhalation exposure to PCBs is well-characterized, and (2) health effects known to be associated with PCB exposure are measured

Exposure assessment

- Congener-specific analyses of PCBs found in air would facilitate comparisons of exposure and effect across studies.
- Use of personal air monitors may reduce uncertainty.
- Data from epidemiological studies conducted to support exposure-response assessment may also support the development of physiologically based pharmacokinetic models, especially if PCBs are measured in biological samples taken at various time points relative to exposure.

Toxicological assessment (examples)

- Serum thyroid hormone levels (e.g., thyroxine and thyroid stimulating hormone) (Schell et al. 2004)
- Susceptibility to infection or antibody responses to immunization as functional measures of immunotoxicity[a] (Weisglas-Kuperus et al. 2000)
- Neurodevelopmental effects in children[a], especially effects on executive function (e.g., response inhibition, attention, working memory and planning) (Jacobson and Jacobson 2003)

Footnote

[a]Both immunotoxicity and neurodevelopmental effects have been associated with PCB exposure in children. Exposure assessment in both children and their mothers during (or even prior to) pregnancy would permit the detection of associations between these endpoints and exposures during sensitive periods of development (Makris et al. 2008).

Exposure-response data from toxicological studies in animals exposed to PCBs by inhalation

Developmental study in monkeys or rats[b]

Footnote

[b]Based on the results of studies utilizing the oral route of exposure, monkeys and rats are sensitive to PCB-induced neurodevelopmental effects (Rice 1999; Sable et al. 2009) and immunotoxicity (Exon et al. 1985; Tryphonas et al. 1991), and rats are sensitive to PCB-induced thyroid hormone disruption (Byrne et al. 1987).

Exposure method[c]

- Exposing dams to airborne PCBs prior to mating and throughout gestation would help to address potential developmental impacts of PCB storage in maternal adipose tissue and partitioning into milk.
- Nose-only inhalation exposure prevents oral exposure resulting from PCB deposition on skin and fur.
- Measurement of health effects at three or more air PCB concentrations may reduce uncertainty in dose-response assessment.

Footnote

[c]Useful data might also be derived from a similar study in which dams are exposed orally to a mixture of PCB congeners similar to what is found in air (e.g. Zhao et al. 2010), especially if toxicokinetic studies are also conducted to inform route-to-route extrapolation.

Exposure assessment

- Careful characterization of the congener profile of the PCB mixture present in the exposure atmosphere would facilitate comparisons of exposure and effect across studies.

- Offspring exposure occurs by multiple routes (e.g., gestational, inhalation, lactational). Measuring PCB body burden in offspring at various time points will facilitate the assessment of total offspring exposure.

Toxicological assessment (examples)

- Cognitive effects measured in offspring, especially effects on executive function
- Immunotoxicity measured in dams and offspring (e.g., impaired antibody response to antigen, decreased natural killer (NK) cell activity)

Chronic or subchronic exposure study in monkeys or rats[b]

<u>Footnote</u>

[b]Based on the results of studies utilizing the oral route of exposure, monkeys and rats are sensitive to PCB-induced neurodevelopmental effects (Rice 1999; Sable et al. 2009) and immunotoxicity (Exon et al. 1985; Tryphonas et al. 1991), and rats are sensitive to PCB-induced thyroid hormone disruption (Byrne et al. 1987).

Exposure method

- Chronic exposure duration: 2 years in rodents (longer in monkeys)
- Subchronic exposure duration: 13 weeks in rodents (longer in monkeys)
- Nose-only inhalation exposure prevents oral exposure resulting from PCB deposition on skin and fur.
- Measurement of health effects at three or more air PCB concentrations may reduce uncertainty in dose-response assessment.

Exposure assessment

- Careful characterization of the congener profile of the PCB mixture present in the exposure atmosphere would facilitate comparisons of exposure and effect across studies.

Toxicological assessment (examples)

- Immunotoxicity measured as impaired antibody response to antigen or as decreased NK cell activity
- Changes in serum thyroid hormone levels

Data to describe the kinetic behavior of inhaled PCBs

Toxicokinetic study in monkeys or rats exposed to PCBs by inhalation

Exposure method

- Nose-only inhalation exposure prevents oral exposure resulting from PCB deposition on skin and fur.

Exposure assessment

- Appropriate interpretation and application of toxicokinetic data may depend on careful characterization of the congener profile of the PCB mixture present in the exposure atmosphere and the congener and metabolite profiles found in excreta and tissue samples collected at various time points relative to exposure.

References

Byrne JJ, Carbone JP, Hanson EA. 1987. Hypothyroidism and abnormalities in the kinetics of thyroid hormone metabolism in rats treated chronically with polychlorinated biphenyl and polybrominated biphenyl. Endocrinology 121:520–527.

Exon JH, Talcott PA, Koller LD. 1985. Effect of lead, polychlorinated biphenyls, and cyclophosphamide on rat natural killer cells, interleukin 2, and antibody synthesis. Fundam Appl Toxicol 5:158–164.

FDA. 2014. Memorandum from Judith Spungen, MS RD to Linda J Phillips, PhD through Deborah Smegal, MPH: Estimated dietary exposure to PCBs based on 2003 Total Diet Study results. Washington, DC:U.S. Department of Health and Human Services, Public Health Service, Food and Drug Administration. Available: http://hero.epa.gov/index.cfm?action=reference.details&reference_id=2346815.

Harrad S, Ibarra C, Robson M, Melymuk L, Zhang X, Diamond M, et al. 2009. Polychlorinated biphenyls in domestic dust from Canada, New Zealand, United Kingdom and United States: Implications for human exposure. Chemosphere 76:232–238.

Jacobson JL, Jacobson SW. 2003. Prenatal exposure to polychlorinated biphenyls and attention at school age. J Pediatr 143:780–788.

Makris SL, Thompson CM, Euling SY, Selevan SG, Sonawane B. 2008. A lifestage-specific approach to hazard and dose-response characterization for children's health risk assessment. Birth Defects Res B Dev Reprod Toxicol 83:530–546.

Motelay-Massei A, Harner T, Shoeib M, Diamond M, Stern G, Rosenberg B. 2005. Using passive air samplers to assess urban-rural trends for persistent organic pollutants and polycyclic aromatic hydrocarbons. 2. Seasonal trends for PAHs, PCBs, and organochlorine pesticides. Environ Sci Technol 39:5763–5773.

Priha E, Hellman S, Sorvari J. 2005. PCB contamination from polysulphide sealants in residential areas-exposure and risk assessment. Chemosphere 59:537–543.

Rice DC. 1999. Behavioral impairment produced by low-level postnatal PCB exposure in monkeys. Environ Res 80:S113–S121.

Roy TA, Hammerstrom K, Schaum J. 2009. Percutaneous absorption of 3,3',4,4'-tetrachlorobiphenyl (PCB 77) from soil. J Toxicol Environ Health A 72:350–357.

Sable HJ, Eubig PA, Powers BE, Wang VC, Schantz SL. 2009. Developmental exposure to PCBs and/or MeHg: Effects on a differential reinforcement of low rates (DRL) operant task before and after amphetamine drug challenge. Neurotoxicol Teratol 31:149–158.

Schell LM, Gallo MV, DeCaprio AP. 2004. Thyroid function in relation to burden of PCBs, p,p'-DDE, HCB, mirex and lead among Akwesasne Mohawk youth: A preliminary study. Environ Toxicol Pharmacol 18:91–99.

Tryphonas H, Luster MI, Schiffman G, Dawson LL, Hodgen M, Germolec D, et al. 1991. Effect of chronic exposure of PCB (Aroclor 1254) on specific and nonspecific immune parameters in the rhesus (Macaca mulatta) monkey. Fundam Appl Toxicol 16:773–786.

U.S. EPA. 2003. Exposure and human health reassessment of 2,3,7,8 tetrachlorodibenzo-p dioxin (TCDD) and related compounds [NAS review draft]. EPA/600/P-00/001. Washington, DC:U.S. Environmental Protection Agency. Available: http://www.epa.gov/nceawww1/pdfs/dioxin/nas-review/

U.S. EPA. 2011. Exposure factors handbook 2011 edition (final). EPA/600/R-09/052F. Washington, DC:U.S. Environmental Protection Agency. Available: http://cfpub.epa.gov/ncea/cfm/recordisplay.cfm?deid=236252

Weisglas-Kuperus N, Patandin S, Berbers GAM, Sas TCJ, Mulder PGH, Sauer PJJ, et al. 2000. Immunologic effects of background exposure to polychlorinated biphenyls and dioxins in Dutch preschool children. Environ Health Perspect 108:1203–1207.

Zhao HX, Adamcakova-Dodd A, Hu D, Hornbuckle KC, Just CL, Robertson LW, et al. 2010. Development of a synthetic PCB mixture resembling the average polychlorinated biphenyl profile in Chicago air. Environ Int 36:819–827.
